# Prostate cancer–Exercise and Metformin Trial (Pre-EMpT): study protocol for a feasibility factorial randomized controlled trial in men with localised or locally advanced prostate cancer

**DOI:** 10.1186/s40814-022-01136-7

**Published:** 2022-08-12

**Authors:** Lucy McGeagh, Luke A. Robles, Raj Persad, Edward Rowe, Amit Bahl, Jonathan Aning, Anthony Koupparis, Paul Abrams, Claire Perks, Jeffrey Holly, Lyndsey Johnson, Constance Shiridzinomwa, Amarnath Challapalli, Ellie Shingler, Hilary Taylor, Jon Oxley, Meda Sandu, Richard M. Martin, J. Athene Lane

**Affiliations:** 1 NIHR Bristol Biomedical Research Centre, University Hospitals Bristol and Weston NHS Foundation Trust and University of Bristol, Bristol, UK; 2 Supportive Cancer Care Research Group, Faculty of Health and Life Sciences, Oxford Institute of Nursing, Midwifery and Allied Health Research, Oxford Brookes University, Oxford, UK; 3 Bristol Urological Institute, North Bristol NHS Trust, Bristol, UK; 4 Bristol Haematology and Oncology Centre, University Hospitals Bristol and Weston NHS Foundation Trust, Bristol, UK; 5 Insulin-like Growth Factors and Metabolic Endocrinology Group, Translational Health Sciences, Bristol Medical School, University of Bristol, Bristol, UK; 6 Clinical Research Centre, North Bristol NHS Trust, Bristol, UK; 7 Bristol Medical School, Population Health Sciences, University of Bristol, Bristol, UK; 8 Department of Cellular Pathology, North Bristol NHS Trust, Bristol, UK

**Keywords:** Prostate cancer, Metformin, Physical activity, Feasibility randomised controlled trial, Localised, Locally advanced, Radiotherapy, Prostatectomy, Active surveillance

## Abstract

**Background:**

Evidence from observational studies have shown that moderate intensity physical activity can reduce risk of progression and cancer-specific mortality in participants with prostate cancer. Epidemiological studies have also shown participants taking metformin to have a reduced risk of prostate cancer. However, data from randomised controlled trials supporting the use of these interventions are limited. The Prostate cancer–Exercise and Metformin Trial examines that feasibility of randomising participants diagnosed with localised or locally advanced prostate cancer to interventions that modify physical activity and blood glucose levels. The primary outcomes are randomisation rates and adherence to the interventions over 6 months. The secondary outcomes include intervention tolerability and retention rates, measures of insulin-like growth factor I, prostate-specific antigen, physical activity, symptom-reporting, and quality of life.

**Methods:**

Participants are randomised in a 2 × 2 factorial design to both a physical activity (brisk walking or control) and a pharmacological (metformin or control) intervention. Participants perform the interventions for 6 months with final measures collected at 12 months follow-up.

**Discussion:**

Our trial will determine whether participants diagnosed with localised or locally advanced prostate cancer, who are scheduled for radical treatments or being monitored for signs of cancer progression, can be randomised to a 6 months physical activity and metformin intervention. The findings from our trial will inform a larger trial powered to examine the clinical benefits of these interventions.

**Trial registration:**

Prostate Cancer Exercise and Metformin Trial (Pre-EMpT) is registered on the ISRCTN registry, reference number ISRCTN13543667. Date of registration 2nd August 2018–retrospectively registered. First participant was recruited on 11th September 2018.

## Background

Prostate cancer is the second commonest cause of UK male cancer death after lung cancer: in 2016, over 11,500 men died as a result of the disease [1]. While 10-year prostate cancer survival in England is high at 78% [1], prostate cancer mortality accelerates after 15 years [2, 3]. Furthermore, men undergoing treatment for early prostate cancer experience adverse effects (e.g. urinary incontinence, sexual dysfunction, fatigue and psychological impacts) [4] and one in three face biochemical recurrence [5]. These data suggest that there may be potential benefits of tertiary prevention interventions (e.g. rehabilitation programs) as an adjunct to primary treatment on long-term mortality and quality of life.

Observational studies suggest that men with prostate cancer who undertake moderate to vigorous physical activity have a reduced risk of biochemical recurrence and mortality from prostate cancer [6, 7]. Specifically, Kenfield et al. [7] observed an inverse association between brisk walking and risk of prostate cancer–specific mortality and brisk walking appears to be acceptable to men who have undergone radical prostatectomy [8]. Potential mechanisms include reduced levels of circulating insulin, insulin-like growth factor-I (IGF-I), and inflammatory cytokines, in turn leading to reduced proliferation and increased apoptosis of prostate cancer cells [9–13]. In addition, a higher body mass index (BMI) is associated with poorer prostate cancer survival rates [14]. A growing body of evidence suggests that physical activity interventions are associated with beneficial outcomes for prostate cancer survivors, such as improved quality of life, reduced fatigue and reduced incontinence [15–17]. Physical activity interventions guided by behaviour change theory [18] and incorporating wearable technology can increase motivation and engagement in physical activity of cancer survivors [19].

Metformin is the first line medication in the glycaemic control of type 2 diabetes [20], but epidemiological studies suggest an inverse association between metformin and risk of prostate and other cancer incidence, biochemical recurrence and mortality [21–23]. It is thought that metformin inhibits cancer cell proliferation via two main mechanisms; directly, through inhibition of the AMPK/mTOR pathway and, indirectly, by lowering systemic insulin levels [21, 24]. Metformin is well tolerated and widely available at a low cost [22, 24].

The Prostate cancer-Exercise and Metformin Trial (Pre-EMpT) is investigating the feasibility of randomising participants diagnosed with localised, or locally advanced, prostate cancer into a 2 × 2 factorial RCT of physical activity and metformin prescription, and assessing whether wrist worn activity trackers increase motivation and improve adherence. The goal is to obtain results that will provide practical information to allow us to develop a larger, fully powered, definitive trial.

## Methods

The methods here will include a description of the COVID-19-related protocol amendments. Pre-EMpT started recruitment in September 2018 and was due to finish in April 2020. In March 2020, recruitment was closed early due to COVID-19, although delivery of the interventions was performed remotely between March and August 2020. A substantial amendment, including amendments to the protocol, was approved by the Health Research Authority (HRA) and Research Ethics Committee (REC) in July 2020.

### Trial design

Pre-EMpT has a multi-level 2 × 2 factorial design. Participants are randomised to both a physical activity level and a metformin level for 6 months. This results in four intervention groups, receipt of (i) both physical activity and metformin; (ii) physical activity only; (iii) metformin only; and (iv) neither intervention, used as the control (reference) group.

### Trial setting and eligibility criteria

Pre-EMpT is being conducted within a large tertiary referral centre in the South West of England. Participants are recruited from urology outpatient clinics. The inclusion criteria are:

- Localised or locally advanced prostate cancer
- Due to undergo external beam radiotherapy/brachytherapy or radical prostatectomy, or due to begin the active surveillance pathway
- Due to receive treatment within the recruiting NHS trust
- Capacity to consent for themselves
- Aged 18 or over
- Have sufficient understanding of the English language
- No contraindication to metformin

Exclusion criteria are the following:

- Inability to give informed consent or unavailability for follow-up
- Being identified as unsuitable to participate by their clinician
- Currently taking metformin or insulin
- Co-morbidities which could prevent metformin prescription
- A physical restriction which would prevent brisk walking

### Consent procedures

This trial employs a 3-part consent process. In the first part (initial consent), participants are consented and randomised over the telephone before their first research clinic appointment so that suitability for metformin can be reviewed and, if not contraindicated, a prescription prepared and dispensed (where necessary) to the research nurse in advance. In the second part (main consent), written informed consent is obtained from each participant when they attend their baseline research clinic appointment. The consent form contains optional clauses to allow the research team to re-contact the participants in relation to further related research, and for their blood and prostate samples to be stored for future (currently unspecified) research purposes. In the third part (interview consent), a proportion of participants are asked to consent to take part in an interview following completion of the active element of the trial after 6 months follow-up.

### Randomisation and blinding

Randomisation will be stratified by treatment group (i.e. external beam radiotherapy/brachytherapy, radical prostatectomy and active surveillance). The research nurse will randomise participants after initial consent, but before main consent, using Research Electronic Data Capture (REDCap, [25, 26]) (a secure web-based system). The research nurse enters the new sequential participant ID and REDCap generates an intervention group for the new participant. This ensures allocation concealment from the nurses who assign participants to the intervention groups.

Participants, and the research team, are not blinded to the intervention allocation. Only those carrying out the analysis are blind to the intervention allocation.

### Interventions

#### Physical activity intervention

Participants allocated to the physical activity group are instructed to walk at a brisk pace for 30 min, on at least 5 days a week, on top of normal physical activity, with the additional aim to walk 10,000 steps every day. They are provided with a waist worn pedometer and a wrist worn Garmin ‘Vivofit 4’ activity monitor. Both the pedometer and Garmin activity monitor will be worn throughout the 6 months duration of the brisk walk intervention. The pedometers measure the number of steps and will be worn throughout the day. The Garmin monitors will be worn day and night and measure number of steps, minutes of moderate physical activity and sleep, and provide movement reminders when an individual has been sedentary for an hour. They can be set to celebrate personalised activity goals and data are available for the individual to view on a smart phone or desktop computer for live feedback. Participants will be asked to record their daily step count for 1 week at baseline, 3, 6, and 12 months follow-up. Physical activity intensity will be recorded by the Garmin activity monitor.

COVID-19 contingency: Participants allocated to brisk walk as part of their intervention will be offered the option of home-based exercises if they are unable, or feel uncomfortable with, brisk walking outside due to COVID-19. Participants will be informed of these exercises via a letter, which will include a 10-min steps workout routine to conduct indoors. This exercise routine is designed to be of similar intensity to brisk walking and safe to perform at home. Participants will be advised that these exercises can be repeated three times a day (30 min in total).

#### Metformin intervention

Participants randomised to the metformin group are prescribed 500 mg slow release metformin per day with food. The tablets are provided at the research clinic visits (baseline and 3 months follow-up).

####  Physical activity and metformin intervention

Participants randomised to the physical activity and metformin group perform both the brisk walking and metformin interventions (as described above) over the 6 months intervention period.

#### Control group

Participants randomised to the control group are asked to carry on with normal levels of physical activity. If participants ask for physical activity advice, standard, publicly available, information would be provided as per usual care.

These participants are not prescribed a placebo for metformin. This study is not designed to test the efficacy of the interventions compared to the control group.

### Strategies to improve adherence to interventions

Participant-facing documents, including the intervention instructions, will be developed in line with the Theory of Planned Behaviour (TPB) [27]. The TPB explores three constructs which predict whether an individual will make a behaviour change or not. These constructs include (1) behavioural beliefs, which produce an attitude towards the behaviour; (2) normative beliefs, which affect perceived social pressure (social norms), and (3) control beliefs which give rise to perceived behavioural control. These constructs come together to influence intention to change behaviour, which can result in change in the actual behaviour. The Garmin activity monitors will also be used as a motivational tool to increase adherence to the brisk walking intervention. These monitors vibrate and display a message when the daily goal of 10,000 steps or weekly goal of 150 moderate intensity minutes is reached, as well as vibrating to notify sedentary activity.

### Harms and provisions for post-trial care

Participants will be informed of the potential risks associated with the interventions before consenting to the trial. These risks include trips or falls and light muscle strain from brisk walking and nausea, diarrhoea, abdominal pain, appetite loss, and taste disturbance from taking metformin. There is no compensation for trial participation.

### Primary and secondary outcomes

Table 1 indicates when each of the primary and secondary outcomes will be collected.

**Table 1:** Pre-EMpT data collection

		Study period						
	Pre-trial	Allocation	Post-allocation					
			RC1		RC2		RC3	
Timepoint		0	~ 3 weeks	~ 9 weeks^a^	3 months	~ 14 weeks^b^	6 months	12 months
**Enrolment:**								
Eligibility screen	x							
Randomisation consent		x						
Informed consent			x					
Interview consent							x	
**Assessments:**								
Demographic data			x		x		x	x
Family medical history			x		x		x	x
Height (cm)			x					
Weight (kg)			x		x		x	
Blood sample			x		(x)		x	
Leisure time physical activity [28]			x		x		x	x
Urinary symptoms [29]			x	x	x		x	x
Psychological factors [30, 31]			x		x		x	x
Patient function and bother [32]			x		x		x	x
Health beliefs [adapted from [33, 34]			x		x		x	x
Quality of life measures [35]			x		x		x	x
Cancer-related fatigue [36, 37]			x		x	x	x	x
Lifestyle behaviours (drinking and smoking)			x		x		x	x
Prostate tissue			[x]					
Step count (pedometer)			x		x		x	x
Physical activity intensity/step count (Garmin)			x		x		x	
Metformin tablet count			x		x		x	
Qualitative interview							x	

*RC* research clinic, *[x]* = collected during surgery 1-week post-baseline; *(x)* = metformin groups only

^a^Surgical participants only

^b^Radiotherapy participants only

#### Primary outcomes

1. Randomisation rate, calculated as the proportion of eligible participants who agree to be randomised for each treatment group. A randomisation rate of 20% or more will be used to determine the feasibility of this outcome.
2. Adherence to the physical activity intervention at 6 months follow-up, assessed via step count recorded by the pedometer for a 1-week period. An adherence rate of 50% or more will be used to determine the feasibility of this intervention based on the same brisk walking intervention in a previously published trial [38].
3. Adherence to the metformin supplementation intervention at 6 months follow-up, measured by tablet count, based on subtracting the number of pills returned at the research clinic visit from the number of pills dispensed. An adherence rate of 60% or more will be used to determine the feasibility of this intervention based on adherence to a nutritional supplementation intervention in a previously published trial [38].

#### Secondary outcomes

1. Feasibility of assessing physical activity levels (self-reported at baseline, 3, 6, and 12 months follow-up and based on the Godin Exercise Leisure-time Questionnaire [28]);
2. Intervention tolerability (qualitatively collected data, reporting of adverse events);
3. Trial retention (number of participants successfully followed-up at the end of the 6 months active intervention element of the trial, as a proportion of those who we recruited to the trial and randomised into a study group at the start of the trial);
4. Feasibility of measuring prostate specific antigen (PSA) level (measured at baseline and 6-months follow-up, collected via blood sample);
5. Feasibility of measuring insulin-like growth factor I (IGF-I) (measured at baseline and 6-months follow-up, collected via blood sample);
6. Feasibility of demonstrating methylation and gene expression profiles in blood (measured via blood and tissue (prostate tissue removed during surgery) as part of standard care) samples at baseline and 6 months follow-up);
7. Urinary symptoms (collected at baseline, 3, 6, and 12 months follow-up and based on the International Continence Society male–Short Form, (ICSmale-SF) [29], and additionally collected at 7 weeks following main consent for participants undergoing surgery);
8. Psychological factors (collected at baseline, 3, 6, and 12 months follow-up and based on the Profile of Mood States (POMS) [30] and Benefit Finding Scale [31]);
9. Patient function and bother after prostate cancer treatment (collected at baseline, 3, 6, and 12 months follow-up and based on the Expanded Prostate Cancer Index Composite (EPIC-26) [32]);
10. Health beliefs (collected at baseline, 3, 6, and 12 months follow-up and based on questionnaires measuring constructs of the transtheoretical model of health behaviour change (i.e. stages of change) [33] and the Theory of Planned Behaviour [34]);
11. Quality of life measures (collected at baseline, 3, 6, and 12 months follow-up and based on the Functional Assessment of Cancer Therapy–Prostate (FACT-P) [35]);
12. Cancer-related fatigue (collected at baseline, 3, 6, and 12 months follow-up and based on the Functional Assessment of Chronic Illness Therapy-Fatigue (FACIT-Fatigue) [36] and the EuroQol 5 dimensions questionnaire (EQ-5D-5L) [37], additionally collected at 4.5 months after main consent for participants undergoing external bean radiotherapy or brachytherapy);
13. General lifestyle factors (self-reported levels of smoking and drinking);
14. Feasibility of participants with localised prostate cancer wearing wrist worn activity trackers (assessed by qualitative interview and the 7 day monitoring form);
15. Impact of wrist worn activity trackers on adherence to the physical activity intervention (assessed by step count on the 7 day monitoring form and data recorded by wrist worn activity trackers);
16. Weight and body mass index (measured by a nurse);
17. Attitudes and views of participants about the physical activity and metformin interventions and participation within the trial (based on qualitative interviews conducted once participants complete 6-months follow-up).

Participants are given the opportunity to complete all questionnaires electronically if they would prefer via a secure online questionnaire system, RedCap.

### Recruitment

All participants diagnosed with prostate cancer at the urology outpatient clinic who meet the eligibility criteria are invited to participate. These participants are identified by the research nurses from all participants with prostate cancer who are discussed at the multi-disciplinary team meeting (MDT) prior to being informed of their diagnosis. At the diagnosis appointment, a clinical nurse specialist introduces the research by saying “We are conducting lifestyle research within the department, are you willing for a research nurse to call you to discuss this further?” If the participant agrees, their contact details are passed on to the research nurse. An informational flyer is available.

The research nurses telephone the potential participants, explain the trial, answer any questions, and confirm eligibility. Eligible and interested participants are then emailed or posted a detailed information sheet. To ensure maximum recruitment, any participants who are missed at the diagnosis clinic are phoned by a member of the clinical care team to explain the trial and provide more information as above. For all participants, the research nurse phones again a few days later once the information has had time to arrive and be read. For those participants still wishing to participate, initial telephone consent is requested for randomisation and preparation of metformin prescriptions (if appropriate). A baseline face to face research appointment is scheduled for approximately 1 week before treatment is due to begin.

Recruitment of participants into the trial will continue until the target sample size of 122 (i.e. *n* = 54, active surveillance; *n* = 40, surgery; *n* = 28, external beam radiotherapy/brachytherapy) has been reached.

### Treatment arm specifics

The trial pathway differs slightly for those undergoing the different treatment pathways.

#### Radical prostatectomy

During radical prostatectomy, participants in the trial have tissue samples extracted and stored. Participants are asked to note how much activity they can do post-surgery and at what stage they feel they are back to their pre-surgery activity. Participants have an additional questionnaire 7 weeks following main consent (approximately 6 weeks after surgery).

#### Active surveillance

As well as being identified from the MDT, active surveillance patients are identified from the cancer register. These participants are contacted 5 to 6 months into the active surveillance pathway, re-screened and sent trial information. Participants on active surveillance undergo a second biopsy, as part of standard care, at 12 months following diagnosis and this recruitment delay allows biopsy data to be timed to be collected after 6 months follow-up.

#### External beam radiotherapy or brachytherapy

Participants undergoing external beam radiotherapy and brachytherapy follow the research pathway as per protocol, with an additional questionnaire at 4.5 months post main consent.

### Research clinic 1: baseline

At the initial research clinic appointment, informed consent is re-confirmed, and the consent form is signed. Baseline data are collected, based on all the outcome measures to be collected at follow-up. Participants are informed of their intervention group and provided with specific intervention group instructions.

The research nurse ensures that the participants know how to carry out their interventions. Participants in the metformin group are seen briefly by a urologist and provided with 3 months medication. All participants are provided with a pedometer to wear continuously for the 6 months duration of the trial. In addition, participants in the physical activity intervention are provided with a Garmin ‘Vivofit 4’ activity tracker to wear for the 6 months duration of the trial intervention. All participants will be given a 7 day monitoring form where they log their daily step count over 1 week and, where appropriate, whether they have completed 10,000 steps, achieved the target brisk walking and taken their metformin tablet. This monitoring form is returned to the trial team by post.

Following baseline research appointment, a letter is sent to the participants’ GP, informing them that their patient is participating in the research trial and letting them know which intervention group they are in.

Approximately 6 weeks after surgery, participants undergoing radical prostatectomy receive an additional urinary symptom questionnaire.

### Research clinic 2: 3 months follow-up

Three months following main consent, and baseline appointment, the participants are sent the questionnaire again, along with a 7 day monitoring form, to be returned by post. The research nurse phones the participants to arrange the 3 months follow-up appointment. Participants will be given the option of performing their follow-up over the telephone, except for participants in the metformin groups who are asked to attend a face-to-face appointment. An additional blood sample is taken from the participants in the metformin groups and their suitability to continue taking metformin is reviewed, and a further 3 months metformin supply is provided.

Approximately 4.5 months post-randomisation, participants undergoing radiotherapy receive an additional symptoms questionnaire including a measure of cancer-related fatigue [36] and quality of life [35].

COVID-19 contingency: participants in the metformin group will have their follow-up appointment conducted over the telephone. Participants with a recent estimated glomerular filtration rate (eGFR) will be administered with a further 3 months supply of metformin either through collecting their metformin in person (with minimal social contact) at the trial site or via use of the hospital pharmacy courier service.

### Research clinic 3: 6 months follow-up

Six months after main consent, participants are sent the questionnaire and 7 day monitoring form to complete before the 6 months follow-up research clinic. At the research clinic, a blood sample is taken, pedometers and wrist worn activity monitors are returned and some participants will be invited to participate in a qualitative interview to explore experiences of the changes they were asked to make and of participating in the research overall.

To engage with the participants and maintain motivation throughout the 6 months intervention period, participants receive texts, emails, and posted materials.

COVID-19 contingency: all participants with have their follow-ups conducted over the telephone. Blood collection will be obtained up to and at 12 months follow-up. Participants will be advised to retain their pedometers for their 12 months follow-up and sent a prepaid envelope to those given a Garmin ‘Vivofit 4’ activity tracker to return the device. The research nurse approached participants about participating in an interview during their telephone follow-up. All interviews will be conducted over the telephone.

### 12 months follow-up

Questionnaire and 7 day monitoring data will be collected at 12 months follow-up via post, 6 months after the intervention has ended.

### Sample size determination and power calculation

The sample size calculation was based on the primary outcome of feasibility of recruitment (assessed by recruitment rate). We aim to recruit 122 participants in total. This equates to 54 participants from the active surveillance group, 40 from the surgical group and 28 from the external beam radiotherapy/brachytherapy group. This allows for an anticipated 20% recruitment rate to be estimated with 95% confidence intervals from 15 to 25%, 14 to 26%, and 14 to 28%, respectively. This trial is not powered to assess any clinical effects, pharmacological effects, identify, verify or compare adverse reactions or study, verify or compare the interventions absorption, distribution, metabolism or excretion. It is not powered to ascertain, verify, or compare the efficacy of the intervention or safety of the intervention.

### Participant timeline

**Fig. 1:**
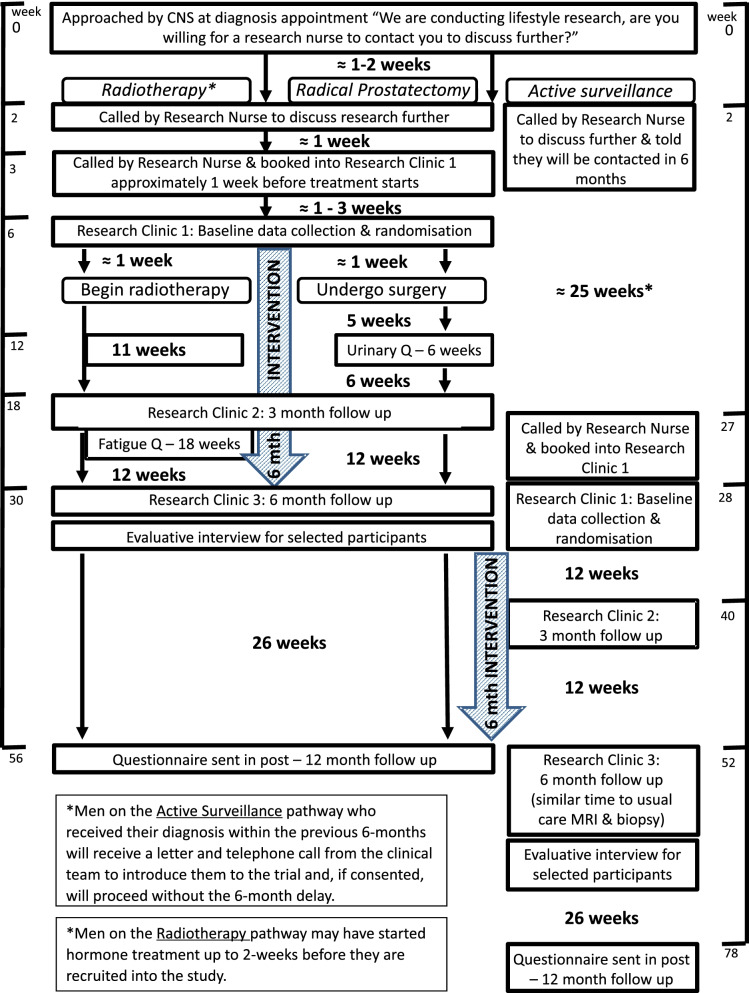
Pre-EMpT participant flow diagram

The participant pathway and data collection through the trial is presented in Fig. 1 and Table 1, respectively.

### Plans for assessment and collection of outcomes

Data will be collected directly from participants with the except of data obtained from medical records (i.e. PSA level) or measured (i.e. height, weight) by the research nurse and reported on case report forms. Data quality will not be performed on the case report form data as the study is designed to assess the feasibility of collecting these data.

### Patient reported outcome measures (PROMs)

All participants will be asked to complete PROMs at baseline, 3, 6, and 12 months follow-up. These PROMs will be used to assess the feasibility of collecting these data from the study sample and will not be used to assess between-group differences. Table 2 provides a description of each of the PROMs.

**Table 2:** Patient reported outcome measures

Questionnaire	Description of measurement	Measurement outcome
Godin Exercise Leisure-time Questionnaire	Number of times of mild, moderate, and strenuous physical activity for more than 15 min per week	Total leisure-time physical activity Categories: Active Moderately active Insufficiently active
International Continence Society male-Short Form (ICSmale-SF)	Urinary symptoms and impact on daily life in the last month	Voiding Incontinence
Profile of Mood States (POMS)	Frequency of 6 mood states over the past week	Tension Depression Anger Fatigue Confusion Vigour
Benefit Finding Scale	Personal growth and acceptance from a diagnosis of prostate cancer	Personal growth Acceptance
The Expanded Prostate Cancer Index Composite (EPIC-26)	Patient function and bother after prostate cancer treatment	Urinary incontinence Urinary obstructive/irritative Bowel Sexual Hormonal
Stages of change questionnaire	Five motivational stages of the transtheoretical model of behaviour change	Precontemplation Contemplators Preparation Action Maintenance
Theory of Planned Behaviour (TPB) questionnaire	Five constructs relating to the TPB	Attitude Perceived norm Perceived behavioural control Intention Past behaviour
Functional Assessment of Cancer Therapy-Prostate (FACT-P)	General health-related and prostate cancer-specific quality of life over the past week	Physical Social/family Emotional Functional Prostate cancer-specific scale
Functional Assessment of Chronic Illness Therapy-Fatigue (FACIT-Fatigue)	Impact of fatigue on daily activities and function over the past week	Overall fatigue score
EQ-5D-5L	Current health status	Mobility Self-care Usual activities Pain/discomfort Anxiety/depression Overall health status

### Tissue and blood sample collection

All human tissue samples are collected, used, and stored in accordance with the Human Tissue Act 2004.

Tumour tissue is extracted as per standard NHS protocols during the prostate biopsy taken at radical prostatectomy (surgical participants). Prostate tissue sections are formalin-fixed and prostate tissue that is surplus to diagnostic requirements will be stored for later research analysis. A small sample of prostate tissue is taken during the radical prostatectomy and transported directly to the study laboratory to be stored in a manner that preserves the genetic material.

Tissue, in the form of plasma, serum and whole blood, will be used to explore the feasibility of assessing the effects of physical activity and metformin interventions on biomarkers of prostate cancer (e.g. expression levels of IGF-I, metabolomic, and epigenetic profiles) using extracted genetic material (DNA/RNA) and the assessment of metabolites and proteins. Blood samples are collected for metabolomics, genetic and epigenetic (DNA, RNA) analysis, and other related research which may be conducted to investigate the feasibility of assessing biological mechanisms linking physical activity, metformin and other factors with prostate cancer risk and progression. Serum blood samples will be processed within 1.5 h from collection to freeze at − 80° centigrade for glycolysis assessment.

### Withdrawal and discontinuation of participants

Withdrawal of participants will be kept to a minimum through regular contact with the participants and ongoing motivational communication. However, participants may be withdrawn from the study by a member of their usual care team or a member of the research team if it is considered detrimental to the participants to continue. Additionally, a participant may withdraw from the study at any time without prejudice to his subsequent treatment. Participants who fail to attend appointments are contacted to encourage them to re-engage with the trial, and, where necessary, to determine reasons for withdrawal. Reasons for withdrawal (if provided) is fully documented, and any adverse events recorded if applicable. If participants switch to a different intervention, post-randomisation, primary outcome data will be analysed as per intention to treat.

### Data management

Personal identifiable data (e.g. names, mailing addresses) collected from the participants will be entered into an electronic database, which will be held securely and separately from other trial data. The database will be hosted by the university for the duration of the research. Access to the database will only be permitted to authorised staff. Anonymous data collected on the case report forms for the research clinic visits, questionnaires, and 7 day monitoring forms will be entered onto REDCap where data will be matched only to participants’ unique identifier. Data entry will be performed by authorised members of staff. Range checks will be performed on numerical values on data collected from the case report forms. Double data entry will be performed on a proportion of the data (e.g. 10%) collected from the questionnaire and 7 day monitoring form. The trial co-ordinator will be responsible for transferring and storing all paper records securely in the Trial office All paper records will be stored in a secure locked cabinet in a locked room and retained for 15 years following completion of the study.

### Confidentiality

Participants will be assigned a unique identifier on enrolled into the trial, which will be used on case report forms and self-report outcome measures to maintain participant anonymity. Personal identifiable data will be entered into an electronic database, which will be held securely and separately from other trial data. Anonymised data will not be shared with other researchers external to the study team.

### Analysis plan

The primary comparative analyses are by intention-to-treat for randomisation rates and adherence to treatment. Baseline characteristics for each group are tabulated using means and standard deviations for normally distributed data, medians and interquartile ranges for non-normally distributed data, and percentages and counts for categorical data. Outcome measures at baseline and follow-up are tabulated to assess the range of values to inform sample size calculations for a definitive trial. Secondary outcomes are tabulated as described above.

The measures made on the tissue and blood samples are research measures and do not have any direct clinical relevance for the participant or their routine clinic treatment. Analysis includes mean serum, plasma, and tissue levels of components of the IGF axis, including IGF-I as a biomarker of prostate cancer progression, and PSA level. Concentrations of IGF-I will be measured using gold standard ‘in-house’ radioimmunoassays. PSA levels will be measured using standard methods employed by the study’s NHS Pathology laboratory. Bloods and tissue may also be analysed for genetic, epigenetic, and metabolomic biomarkers.

Semi-structured face-to-face or telephone interviews will be conducted with participants to explore their experiences of their allocated intervention and the trial generally. Purposive sampling will be used to recruit up to 5 participants from each of the interventions, who underwent different treatment types and who wore the wearable activity tracker. The interviews will be audio-recorded, transcribed, anonymised, and analysed thematically using a qualitative method. A qualitative analysis computer program, such as NVivo [39], will be used to assist with the analysis.

### Composition of the coordinating centre

The day-to-day running of the trial will be supported by the Trial Management Group (TMG), which will be comprised of the Chief Investigators and the trial co-ordinator. The TMG will meet approximately once a month to monitor recruitment and data collection of the trial. We do not have a Trial Steering Committee. However, the Bristol Biomedical Research Centre management group, which includes workstream leads and management of the theme, will provide semi-independent oversight by reviewing recruitment and progress with the trial.

A prostate cancer Public and Patient Involvement group (PPI) has been involved in the development and delivery of Pre-EMpT. The group consists of participants who have or have previously had prostate cancer. They have been trained in different types of research methods and participant facing documents and they meet on a regular basis to discuss different aspects of the trial. This includes reviewing details of the interventions, testing data collection methods, and providing feedback on participant literature. If the trial experiences issues, for example, with recruitment, the group will explore possible barriers and solutions.

### Safety reporting

The Summary of Product Characteristics for Metformin (500 mg) is used as the Reference Safety Information for this trial. Adverse events (AEs) resulting from the intervention are recorded by the nurse or member of the research team; if an event is classified as serious (SAE), an assessment is made by the chief investigator as to whether the event is related to the study intervention and an 'unexpected' event. If an event is both related and unexpected it becomes a Suspected Unexpected Serious Adverse Reaction (SUSAR). These events are reported to the Sponsor immediately, and subsequent protocols followed.

The trial will be monitored in accordance with University Hospitals Bristol and Weston NHS Foundation Trust Monitoring and Oversight of Research Activity Standard Operating Procedure. The monitoring plan will be developed and agreed by the sponsor.

## Discussion

This study aims to investigate the feasibility of randomising participants diagnosed with localised or locally advanced prostate cancer to a physical activity and metformin supplementation intervention. Results of the primary outcomes of this study will be used to determine progression to a definitive RCT assessing the efficacy of the interventions compared to controls in participants with prostate cancer.

### Dissemination policy

We do not have permission to share the anonymised datasets with researchers external to the study team. Results from the trial will be disseminated in presentations at scientific meetings and publication of findings in scientific literature. A summary of the final results of the trial will be available for all participants.

### Trial status

Protocol V6 18/06/2020. Recruitment began on 11th september 2018 and finished on 16th march 2020. Pre-EMpT recruited 110 participants at time of submission. We anticipate that data collection will finish 31st March 2021.

## Data Availability

Any data required to support the protocol can be supplied on request.

## Abbreviations

AE: Adverse event
BMI: Body mass index
CRUK: Cancer Research UK
eGFR: Estimated glomerular filtration rate
FACIT–Fatigue: Functional Assessment of Chronic Illness Therapy-Fatigue
FACT-P: Functional Assessment of Cancer Therapy–Prostate
HRA: Health Research Authority
ICSmale-SF: International Continence Society male–Short Form
IGF-I: Insulin-like growth factor I
NHS: National Health Service
POMS: Profile of Mood States–Short Form
Pre-EMpT: Prostate cancer–Exercise and Metformin Trial
PSA: Prostate specific antigen
R&D: Research and Development
RCT: Randomised control trial
REC: Research Ethics Committee
SAE: Serious adverse event

## References

[1] CRUK. Prostate cancer statistics. https://www.cancerresearchuk.org/health-professional/cancer-statistics/statistics-by-cancer-type/prostate-cancer. Accessed 19 June 2017.

[2] Albertsen PC, Hanley JA, Fine J (2005). 20-year outcomes following conservative management of clinically localized prostate cancer. JAMA..

[3] Johansson JE, Andrén O, Andersson SO, Dickman PW, Holmberg L, Magnuson A (2004). Natural history of early, localized prostate cancer. Jama..

[4] Donovan JL, Hamdy FC, Lane JA, Mason M, Metcalfe C, Walsh E (2016). Patient-reported outcomes after monitoring, surgery, or radiotherapy for prostate cancer. N Engl J Med.

[5] Rosenbaum E, Partin A, Eisenberger MA (2004). Biochemical relapse after primary treatment for prostate cancer: studies on natural history and therapeutic considerations. J Natl Compr Cancer Netw.

[6] Moore SC, Peters TM, Ahn J, Park Y, Schatzkin A, Albanes D (2008). Physical activity in relation to total, advanced, and fatal prostate cancer. Cancer Epidemiol Biomark Prev.

[7] Kenfield SA, Stampfer MJ, Giovannucci E, Chan JM (2011). Physical activity and survival after prostate cancer diagnosis in the health professionals follow-up study. J Clin Oncol..

[8] Shingler E, Hackshaw-McGeagh L, Robles L, Persad R, Koupparis A, Rowe E (2017). The feasibility of the Prostate cancer: Evidence of Exercise and Nutrition Trial (PrEvENT) dietary and physical activity modifications: a qualitative study. Trials..

[9] Barnard RJ, Ngo TH, Leung PS, Aronson WJ, Golding LA (2003). A low-fat diet and/or strenuous exercise alters the IGF axis in vivo and reduces prostate tumor cell growth in vitro. Prostate.

[10] Barb D, Williams CJ, Neuwirth AK, Mantzoros CS (2007). Adiponectin in relation to malignancies: a review of existing basic research and clinical evidence. Am J Clin Nutr.

[11] Haverkamp J, Charbonneau B, Ratliff TL (2008). Prostate inflammation and its potential impact on prostate cancer: a current review. J Cell Biochem.

[12] Frasca F, Pandini G, Sciacca L, Pezzino V, Squatrito S, Belfiore A (2008). The role of insulin receptors and IGF-I receptors in cancer and other diseases. Arch Physiol Biochem.

[13] Lee IM (2008). Epidemiologic methods in physical activity studies.

[14] Calle EE, Rodriguez C, Walker-Thurmond K, Thun MJ (2003). Overweight, obesity, and mortality from cancer in a prospectively studied cohort of U.S. adults. N Engl J Med.

[15] Baumann FT, Zopf EM, Bloch W (2012). Clinical exercise interventions in prostate cancer patients—a systematic review of randomized controlled trials. Support Care Cancer.

[16] Bourke L, Smith D, Steed L, Hooper R, Carter A, Catto J (2016). Exercise for Men with Prostate Cancer: A Systematic Review and Meta-analysis. Eur Urol.

[17] Larkin D, Lopez V, Aromataris E (2014). Managing cancer-related fatigue in men with prostate cancer: A systematic review of non-pharmacological interventions. Int J Nurs Pract.

[18] Grimmett C, Corbett T, Brunet J, Shepherd J, Pinto BM, May CR, Foster C (2019). Systematic review and meta-analysis of maintenance of physical activity behaviour change in cancer survivors. Int J Behav Nutr Phys Act.

[19] Gell NM, Grover KW, Humble M, Sexton M, Dittus K (2017). Efficacy, feasibility, and acceptability of a novel technology-based intervention to support physical activity in cancer survivors. Support Care Cancer.

[20] NICE. Type 2 diabetes in adults: management. NICE guideline [NG28]. 2015. https://www.nice.org.uk/guidance/ng28/chapter/Recommendations. Accessed 20 June 2020.

[21] Whitburn J, Edwards CM, Sooriakumaran P (2017). Metformin and prostate cancer: a new role for an old drug. Curr Urol Rep.

[22] Stopsack KH, Ziehr DR, Rider JR, Giovannucci EL (2016). Metformin and prostate cancer mortality: a meta-analysis. Cancer Causes Control.

[23] Zi F, Zi H, Li Y, He J, Shi Q, Cai Z (2018). Metformin and cancer: An existing drug for cancer prevention and therapy. Oncol Lett.

[24] Coyle C, Cafferty FH, Vale C, Langley RE (2016). Metformin as an adjuvant treatment for cancer: a systematic review and meta-analysis. Ann Oncol.

[25] Harris PA, Taylor R, Thielke R, Payne J, Gonzalez N, Conde JG (2009). Research electronic data capture (REDCap)--a metadata-driven methodology and workflow process for providing translational research informatics support. J Biomed Inform.

[26] Harris PA, Taylor R, Minor BL, Elliott V, Fernandez M, O'Neal L (2019). The REDCap consortium: Building an international community of software platform partners. J Biomed Inform.

[27] Ajzen I (1991). The theory of planned behavior. Organ Behav Hum.

[28] Godin G, Shephard RJ (1985). A simple method to assess exercise behavior in the community. Can J Appl Sport Sci.

[29] Donovan JL, Peters TJ, Abrams P, Brookes ST, de aa Rosette JJ, Schafer W (2000). Scoring the short form ICSmaleSF questionnaire. International Continence Society. J Urol.

[30] McNair DM, Lorr M, and Droppleman LF (1971). Profile of Mood States Manual.

[31] Antoni MH, Lehman JM, Kilbourn KM, Boyers AE, Culver JL, Alferi SM (2001). Cognitive-behavioral stress management intervention decreases the prevalence of depression and enhances benefit finding among women under treatment for early-stage breast cancer. Health Psychol.

[32] Wei JT, Dunn RL, Litwin MS, Sandler HM, Sanda MG (2000). Development and validation of the expanded prostate cancer index composite (EPIC) for comprehensive assessment of health-related quality of life in men with prostate cancer. Urology..

[33] Prochaska JO, DiClemente CC (1983). Stages and processes of self-change of smoking: Toward an integrative model of change. J Consult Clin Psychol.

[34] Fishbein M, Ajzen I (2010). Predicting and changing behavior: The reasoned action approach.

[35] Esper P, Mo F, Chodak G, Sinner M, Cella D, Pienta KJ (1997). Measuring quality of life in men with prostate cancer using the functional assessment of cancer therapy-prostate instrument. Urology..

[36] Yellen SB, Cella DF, Webster K, Blendowski C, Kaplan E (1997). Measuring fatigue and other anemia-related symptoms with the Functional Assessment of Cancer Therapy (FACT) measurement system. J Pain Symptom Manag.

[37] Rabin R, Charro F (2001). EQ-SD: a measure of health status from the EuroQol Group. Ann Med.

[38] Hackshaw-McGeagh LE, Penfold C, Shingler E, Robles LA, Perks CM, Holly JM, Rowe E, Koupparis A, Bahl A, Persad R, Shiridzinomwa C (2019). Phase II randomised control feasibility trial of a nutrition and physical activity intervention after radical prostatectomy for prostate cancer. BMJ Open.

[39] NVivo 12. QSR International. https://www.qsrinternational.com/nvivo-qualitative-data-analysis-software/home. Accessed 25 Nov 2021.

